# Long-term Impact of Cardiorespiratory Fitness on Type 2 Diabetes Incidence: A Cohort Study of Japanese Men

**DOI:** 10.2188/jea.JE20170017

**Published:** 2018-05-05

**Authors:** Ryoko Kawakami, Susumu S. Sawada, I-Min Lee, Yuko Gando, Haruki Momma, Shin Terada, Chihiro Kinugawa, Takashi Okamoto, Koji Tsukamoto, Mitsuru Higuchi, Motohiko Miyachi, Steven N. Blair

**Affiliations:** 1 Faculty of Sport Sciences, Waseda University, Tokorozawa, Saitama, Japan; 2 Department of Health Promotion and Exercise, National Institutes of Biomedical Innovation, Health and Nutrition, Tokyo, Japan; 3 Division of Preventive Medicine, Brigham and Women’s Hospital, Harvard Medical School, Boston, MA, United States; 4 Department of Epidemiology, Harvard T.H. Chan School of Public Health, Boston, MA, United States; 5 Division of Biomedical Engineering for Health and Welfare, Tohoku University Graduate School of Biomedical Engineering, Sendai, Japan; 6 Department of Life Sciences, Graduate School of Arts and Sciences, The University of Tokyo, Tokyo, Japan; 7 Health Promotion Center, Tokyo Gas Co., Ltd., Tokyo, Japan; 8 Arnold School of Public Health, University of South Carolina, Columbia, SC, United States

**Keywords:** hyperglycemia, physical fitness, exercise test, epidemiology, cohort study

## Abstract

**Background:**

We sought to examine the association between cardiorespiratory fitness (CRF) and incidence of type 2 diabetes considering the follow-up period in a cohort of Japanese men with a maximum follow-up period of 23 years.

**Methods:**

This study enrolled 7,804 male workers free of diabetes in 1986. CRF was measured using a cycle ergometer, and maximal oxygen uptake was estimated. During 1986–2009, participants were followed for development of type 2 diabetes, which was diagnosed using fasting blood tests, self-administered questionnaires, or oral glucose tolerance tests after urinary tests from annual health checkups. Hazard ratios for the incidence of type 2 diabetes were estimated using Cox proportional hazards models.

**Results:**

During the follow-up period, 1,047 men developed type 2 diabetes. In analyses by follow-up periods (1986–1993, 1994–2001, and 2002–2009), there was an inverse dose-response relationship between CRF and the development of type 2 diabetes for all three follow-up periods (*P* for trend 0.019, <0.001, and 0.001, respectively), and the association between CRF at baseline and the incidence of type 2 diabetes did not weaken with longer follow-up period. Compared with the lowest CRF group, hazard ratios of developing type 2 diabetes were 0.69 (95% confidence interval [CI], 0.49–0.97) for the highest CRF group in 1986–1993, 0.57 (95% CI, 0.42–0.79) for the highest CRF in 1994–2001, and 0.47 (95% CI, 0.30–0.74) for the highest CRF in 2002–2009.

**Conclusion:**

High CRF is associated with a lower risk of the incidence of type 2 diabetes over an extended period of >20 years among men.

## INTRODUCTION

The number of patients with diabetes continues to rise across the globe, and has been estimated to increase from 415 million people in 2015 to 642 million people by 2040 according to the International Diabetes Federation.^1^

The results of previous cohort studies^2^^–^^9^ and meta-analyses^10^^,^^11^ have indicated an inverse association between cardiorespiratory fitness (CRF) and the incidence of type 2 diabetes. CRF is generally known to decrease with age,^12^^,^^13^ but reports suggest that CRF can be increased even in middle age by exercise training.^14^ That is, CRF is an indicator that can change in many ways, not only due to aging but also because of intentional or unintentional changes in lifestyle habits and living environments. Cohort studies examining the relationships between changes in CRF and subsequent type 2 diabetes incidence have reported an association between increasing CRF with a lower incidence rate of type 2 diabetes over periods ranging from 13 to 14 years.^15^^,^^16^ Therefore, the influence of CRF level at baseline on subsequent type 2 diabetes incidence can be assumed to weaken due to the impact of aging and changes in lifestyle habits and living environments. On the other hand, exposure to a high CRF at some time may cause epigenetic modifications and be stored as metabolic memory in the body over an extended period.^17^ It is possible that this CRF level at baseline may influence future type 2 diabetes incidence over a prolonged period.

However, to our knowledge, there have been no longitudinal studies regarding the follow-up period for CRF level at any one point in time and subsequent period up to which CRF is associated with type 2 diabetes incidence. Therefore, the present study was performed to examine the association between CRF and type 2 diabetes incidence considering the follow-up period in a cohort of Japanese men with a maximum follow-up period of 23 years.

## METHODS

### Study population

This research is based on a prospective cohort study investigating the relationship between CRF and health outcomes in Japanese males.^4^^,^^6^^,^^16^^,^^18^^,^^19^ Participants in this study were employees of a company based in the Tokyo area of Japan. All employees received annual health checkups and an exercise test once per year, with the aim of managing the health of employees under the Industrial Safety and Health Act and related laws in Japan.

Participants comprised 9,221 employees who underwent an annual health checkup and exercise test from April 1986 through March 1987. We excluded 25 employees who could not continue the exercise test for at least 4 min due to the appearance of abnormal electrocardiogram results or poor physical condition and for whom accurate CRF could therefore not be measured. Due to the small number of female participants (*n* = 790), women were also excluded from this study. In addition, nine employees with cardiovascular disease, three employees with a history of stroke, and 33 employees with diabetes as of 1986 were excluded. Of the remaining 8,361 participants, 197 who could not be followed-up because they retired by 1987, and 360 employees with missing covariate data were also excluded. Ultimately, 7,804 men aged from 19 to 60 years were followed-up until 2009 as participants in this study.

In our observational study, the clinical examinations were done under the Industrial Safety and Health Act and related laws in Japan. Therefore, we did not need to get written informed consent. This study was approved by the ethics committee of the National Institutes of Biomedical Innovation, Health and Nutrition.

### Assessment of cardiorespiratory fitness

Maximum oxygen uptake (${\dot{\text{V}}\text{O}}_{\text{2}}\text{max}$), an indicator for CRF, was estimated in the participants in this study by means of a submaximal exercise test using a cycle ergometer. The exercise test was composed of a maximum of three stages, each lasting 4 min. As the stage proceeded, the load also gradually increased. Load upon starting the test was 98, 86, 74, and 61 W for participants aged 19–29, 30–39, 40–49, and 50–60 years, respectively. Heart rate was measured using the R-R interval on an electrocardiogram. Target heart rate was set as 85% of maximum heart rate estimated from age (220 − age) and load was gradually added in increments of 37 W for each stage until the participant reached the target heart rate. When abnormal electrocardiogram results, such as increased premature ventricular contraction, were observed during exercise testing, the exercise test was discontinued prematurely. ${\dot{\text{V}}\text{O}}_{\text{2}}\text{max}$ was estimated using the Åstrand-Ryhming nomogram,^20^ based on heart rate obtained from the last 1 minute of the final stage of each participant, and the Åstrand age-correction factors.^21^ The method of estimating ${\dot{\text{V}}\text{O}}_{\text{2}}\text{max}$ used in this study has been shown to strongly correlate with results determined using a direct method according to comparative studies.^22^^,^^23^

### Diagnosis of type 2 diabetes

We determined the year that participants developed type 2 diabetes based on the results of annual health checkups made from 1986 through 2009. Between 1986 and 1987, urine glucose testing and 75 g oral glucose tolerance testing in health checkups were used to determine the year that the employee had developed type 2 diabetes. If employees aged ≥40 years had a positive urine glucose test, re-testing was performed at an in-house hospital. If they tested positive again, an oral glucose tolerance test was then performed. The year in which blood glucose levels 2 hours after glucose loading were ≥200 mg/dL (11.1 mmol/L) was considered to be the year that the employee developed type 2 diabetes. If employees aged <40 years had a positive urine glucose test, they were advised to undergo repeat testing of urine glucose at an external hospital. From 1988 onward, fasting blood glucose levels were used to determine the year in which employees developed type 2 diabetes. From 1988 through 1993, blood testing was performed on employees aged 35 years and those aged ≥40 years. From 1994, blood testing was performed on employees aged 25, 30, 35, and ≥40 years. Employees who underwent such blood testing were instructed to fast for 12 hours prior to the blood test. Although it was confirmed when performing the blood test whether the employee had complied with this fasting requirement, there is a possibility that some employees did eat something but did not declare it or forgot to declare it. Therefore, we considered employees for whom fasting blood glucose levels exceeded 126 mg/dL (7.0 mmol/L) twice or more in 6 years to have type 2 diabetes. The year in which fasting blood glucose was first observed to be ≥126 mg/dL was considered to be the year in which type 2 diabetes was developed. From 2007, hemoglobin A1c (HbA1c; NGSP) levels were also used to determine the year in which type 2 diabetes was developed. As with fasting blood glucose levels, when employees exhibited HbA1c levels of ≥6.5% (48 mmol/mol) twice or more in 6 years, the year in which this level was first observed was considered to be the year in which they had developed type 2 diabetes. Blood glucose and HbA1c levels used to diagnose type 2 diabetes were set according to guidelines of the American Diabetes Association^24^ and the Japan Diabetes Society.^25^ In the health checkups during all periods from 1989 through 2009, self-administered questionnaires were used to determine employees’ treatment status for conditions, if any, and whether they had been diagnosed with type 2 diabetes at an external hospital. When there were multiple cases for which the above determination criteria applied, the year in which the earliest case was confirmed was considered to be the year in which the employee had developed type 2 diabetes.

### Assessment of potential risk factors

The height, weight, and resting blood pressure of participants in this study were measured in an annual health checkup performed in 1986. Weight was measured using a set of scales that were regularly inspected according to the law, with employees wearing light clothing and no shoes. Body mass index (BMI) was calculated based on measurement results for height and weight [weight (kg) divided by height (m)^2^]. Resting blood pressure was measured with employees seated on a chair and using an automated sphygmomanometer. Self-administered questionnaires were also used to investigate smoking habits (non-smoker, past smoker, 1–20 cigarettes/day, or ≥21 cigarettes/day), drinking habits (none, 1–20 g/day, or ≥21 g/day), and family history of diabetes (yes or no). Employees were determined to have a family history of diabetes if they answered so at least once in the questionnaire used during annual health checkups from 1986 through 2009.

### Statistical analysis

Participants were classified into quartiles according to CRF by age group (≤24, 25–29, 30–34, 35–39, 40–44, 45–49, and ≥50 years). Continuous variable data are shown as mean (standard deviation) and categorical variable data are shown as percentages.

To investigate the relationship between CRF and type 2 diabetes incidence, Cox proportional hazards regression analyses were performed using the presence of type 2 diabetes as the dependent variable and CRF quartile as the independent variable. We then calculated age-adjusted hazard ratios and 95% confidence intervals. In adjusted models, we calculated hazard ratios with adjustment for age (continuous variable), BMI (continuous variable), systolic blood pressure (continuous variable), smoking habit (non-smoker, past smoking, 1–20 cigarettes/day, or ≥21 cigarettes/day), drinking habit (none, 1–20 g/day, or ≥21 g/day), and family history of diabetes (yes or no).

To investigate the effect of time period on the association between type 2 diabetes and CRF, we calculated *P* values for interaction between duration of follow-up period and CRF on incidence of type 2 diabetes using a time-varying covariate in a Cox proportional hazards model adjusted for potential risk factors (age, BMI, systolic blood pressure, smoking habit, drinking habit, and family history of diabetes). In addition, hazard ratios for type 2 diabetes were calculated each for three follow-up periods: 1986–1993, 1994–2001, and 2002–2009. For analyses of the 1994–2001 period, 6,765 employees (378 employees who developed type 2 diabetes by 1993 and 661 employees who dropped out by 1993 were excluded) were classified into quartiles based on CRF in an age-specific manner. For analyses of the 2002–2009 period, 5,020 employees (799 employees who developed type 2 diabetes by 2001 and 1,985 employees who dropped out by 2001 were excluded) were classified into quartiles based on CRF in an age-specific manner.

We also performed stratified analyses in the 2002–2009 follow-up period based on the potential risk factors of age, BMI, blood pressure, smoking habit, drinking habit, and family history of diabetes to evaluate whether CRF still related with type 2 diabetes independent from other potential risk factors after a long period of time had passed from the start of the follow-up.

We confirmed, using log-minus-log plots, that the proportional hazards assumption was met. All statistical analyses were performed using SPSS Statistics version 23 (IBM Corporation, Armonk, NY, USA). A statistically significant difference was considered to be present when the two-tailed *P* value was <0.05.

## RESULTS

Table 1 shows the characteristics of 7,804 participants at baseline in 1986. The mean age at baseline was 38 years. Mean ${\dot{\text{V}}\text{O}}_{\text{2}}\text{max}$, which was used as an indicator for CRF, was 39.1 mL/kg/min. In the highest CRF group (quartile 4), BMI and systolic and diastolic blood pressure levels were low, and there were fewer smokers. With regards to drinking habit and family history of diabetes, no clear differences were observed between CRF categories.

**Table 1.** Baseline characteristics according to cardiorespiratory fitness category (*n* = 7,804)

	Overall		Cardiorespiratory fitness category							

			Quartile 1		Quartile 2		Quartile 3		Quartile 4	
	(*n* = 7,804)		(*n* = 2,157)		(*n* = 1,972)		(*n* = 1,871)		(*n* = 1,804)	
${\dot{\text{V}}\text{O}}_{\text{2}}\text{max}$, mL/kg/min	39.1	(8.5)	30.6	(4.2)	36.4	(3.5)	41.5	(4.2)	49.8	(6.8)
Age, years	38	(10)	38	(10)	39	(10)	37	(9)	37	(10)
Height, cm	168.6	(5.7)	169.1	(5.8)	168.7	(5.7)	168.3	(5.5)	168.1	(5.7)
Weight, kg	65.9	(8.4)	69.4	(9.1)	66.5	(8.1)	64.3	(7.5)	62.7	(6.9)
Body mass index, kg/m^2^	23.2	(2.6)	24.2	(2.8)	23.4	(2.5)	22.7	(2.4)	22.2	(2.1)
Systolic blood pressure, mm Hg	128.8	(13.3)	132.7	(13.1)	129.7	(12.6)	127.0	(13.0)	124.9	(13.0)
Diastolic blood pressure, mm Hg	74.2	(9.0)	77.0	(9.1)	75.0	(8.6)	72.7	(8.7)	71.4	(8.6)
Smokers, %	62.6		67.0		62.9		61.5		58.3	
Drinkers, %	71.1		70.4		70.2		72.2		71.8	
Family history of diabetes, %	30.1		31.6		30.2		29.3		29.1	

Data are expressed as mean (standard deviation) or %.

During the follow-up period with a maximum of 23 years from 1986 through 2009 (median follow-up period of 19 years, 130,996 man-years), type 2 diabetes developed in 1,047 participants (13.4%). The mean age at time of developing type 2 diabetes was 48 years. The number of type 2 diabetes patients was 378 (4.8%) in 1986–1993, 421 (6.2%) in 1994–2001, and 248 (4.9%) in 2002–2009.

**Figure 1.**
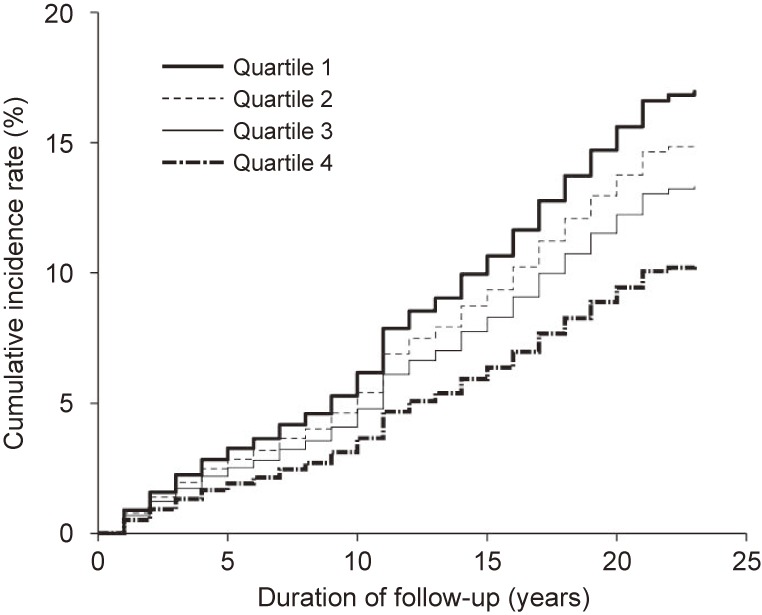
Multivariable-adjusted cumulative incidence curve for type 2 diabetes according to cardiorespiratory fitness category in 1986–2009. Adjusted for age (continuous variable), body mass index (continuous variable), systolic blood pressure (continuous variable), smoking habit (non-smoker, past smoking, 1–20 cigarettes/day, or ≥21 cigarettes/day), drinking habit (none, 1–20 g/day, or ≥21 g/day), and family history of diabetes (yes or no).

Table 2 shows the hazard ratios and 95% confidence intervals for the development of type 2 diabetes according to CRF category. After adjusting for age, systolic blood pressure, smoking habit, drinking habit, and family history of diabetes (model 1), a significant inverse dose-response relationship was observed between CRF and type 2 diabetes incidence (*P* for trend <0.001). After further adjustment for BMI (model 2), the relationship between CRF and type 2 diabetes incidence remained statistically significant (*P* for trend <0.001). Figure 1 shows the multivariate-adjusted cumulative incidence curve for type 2 diabetes according to CRF category.

**Table 2.** Hazard ratios (95% confidence intervals) for incidence of type 2 diabetes according to cardiorespiratory fitness category, 1986–2009 (*n* = 7,804)

	Cardiorespiratory fitness category				*P* trend

	Quartile 1	Quartile 2	Quartile 3	Quartile 4	
*n*	2,157	1,972	1,871	1,804	
Man-years	34,536	32,298	32,834	31,328	
Cases	412	289	213	133	
Age-adjusted model	1.00 (Reference)	0.72 (0.62–0.84)	0.55 (0.47–0.65)	0.35 (0.29–0.43)	<0.001
Multivariable model 1	1.00 (Reference)	0.78 (0.67–0.91)	0.63 (0.54–0.75)	0.43 (0.35–0.52)	<0.001
Multivariable model 2	1.00 (Reference)	0.87 (0.75–1.02)	0.77 (0.65–0.91)	0.58 (0.48–0.72)	<0.001

Multivariable model 1 was adjusted for age (continuous variable), systolic blood pressure (continuous variable), smoking habit (non-smoker, past smoking, 1–20 cigarettes/day, or ≥21 cigarettes/day), drinking habit (none, 1–20 g/day, or ≥21 g/day), and family history of diabetes (yes or no).

Multivariable model 2 was adjusted for model 1 covariates plus body mass index (continuous variable).

**Figure 2.**
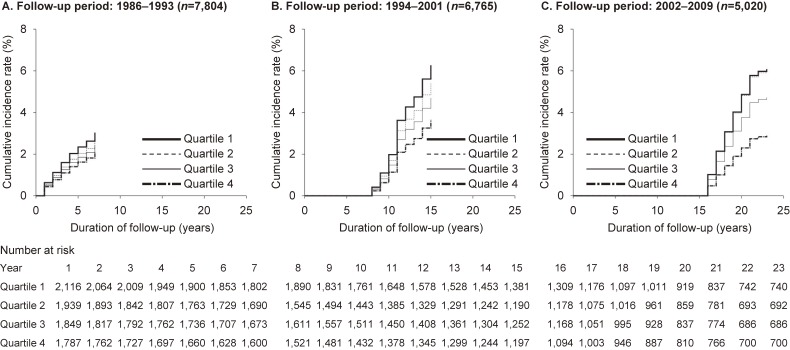
Multivariable-adjusted cumulative incidence curve for type 2 diabetes according to cardiorespiratory fitness category by follow-up period (1986–1993 (A), 1994–2001 (B), and 2002–2009 (C)). Adjusted for age (continuous variable), body mass index (continuous variable), systolic blood pressure (continuous variable), smoking habit (non-smoker, past smoking, 1–20 cigarettes/day, or ≥21 cigarettes/day), drinking habit (none, 1–20 g/day, or ≥21 g/day), and family history of diabetes (yes or no).

To evaluate the relationship between CRF and type 2 diabetes incidence by follow-up period, we calculated *P* values for interaction between duration of follow-up period and CRF on incidence of type 2 diabetes. There were no interaction between duration of follow-up period and CRF (duration of follow-up: ≤7 years vs >7 years [*P* for interaction = 0.630], duration of follow-up: ≤15 years vs >15 years [*P* for interaction = 0.524]). We also calculated the type 2 diabetes hazard ratio and 95% confidence interval according to the CRF category for each follow-up period (Table 3). After adjusting for potential risk factors, we observed a significant, inverse dose-response relationship between CRF and the development of type 2 diabetes for all the follow-up periods of 1986–1993, 1994–2001, and 2002–2009. Figure 2 also shows the multivariate-adjusted cumulative incidence curve for type 2 diabetes according to CRF category for each follow-up period. These results demonstrated that the significant, inverse dose-response relationship between CRF and type 2 diabetes incidence was present not only for the short-term period after starting follow-up, but also over longer-term follow-up periods.

**Table 3.** Hazard ratios (95% confidence intervals) for incidence of type 2 diabetes according to cardiorespiratory fitness category by follow-up period (1986–1993, 1994–2001, and 2002–2009)

	Cardiorespiratory fitness category				*P* trend

	Quartile 1	Quartile 2	Quartile 3	Quartile 4	
1986–1993 (*n* = 7,804)					
*n*	2,157	1,972	1,871	1,804	
Man-years	14,048	12,945	12,534	12,065	
Cases	159	103	68	48	
Age-adjusted model	1.00 (Reference)	0.67 (0.52–0.85)	0.51 (0.38–0.68)	0.36 (0.26–0.49)	<0.001
Multivariable model 1	1.00 (Reference)	0.75 (0.59–0.97)	0.61 (0.46–0.81)	0.45 (0.32–0.63)	<0.001
Multivariable model 2	1.00 (Reference)	0.87 (0.67–1.11)	0.79 (0.59–1.06)	0.69 (0.49–0.97)	0.019
1994–2001 (*n* = 6,765)^a^					
*n*	1,963	1,597	1,644	1,561	
Man-years	27,393	22,505	23,354	22,188	
Cases	176	106	85	54	
Age-adjusted model	1.00 (Reference)	0.73 (0.57–0.93)	0.56 (0.43–0.73)	0.38 (0.28–0.51)	<0.001
Multivariable model 1	1.00 (Reference)	0.79 (0.62–1.00)	0.64 (0.49–0.83)	0.45 (0.33–0.61)	<0.001
Multivariable model 2	1.00 (Reference)	0.86 (0.67–1.10)	0.74 (0.57–0.97)	0.57 (0.42–0.79)	<0.001
2002–2009 (*n* = 5,020)^b^					
*n*	1,398	1,251	1,219	1,152	
Man-years	29,459	26,579	25,943	24,638	
Cases	97	74	51	26	
Age-adjusted model	1.00 (Reference)	0.83 (0.62–1.13)	0.59 (0.42–0.83)	0.32 (0.21–0.49)	<0.001
Multivariable model 1	1.00 (Reference)	0.88 (0.65–1.19)	0.65 (0.46–0.92)	0.36 (0.23–0.57)	<0.001
Multivariable model 2	1.00 (Reference)	0.99 (0.73–1.34)	0.77 (0.54–1.09)	0.47 (0.30–0.74)	0.001

Multivariable model 1 was adjusted for age (continuous variable), systolic blood pressure (continuous variable), smoking habit (non-smoker, past smoking, 1–20 cigarettes/day, or ≥21 cigarettes/day), drinking habit (none, 1–20 g/day, or ≥21 g/day), and family history of diabetes (yes or no).

Multivariable model 2 was adjusted for model 1 covariates plus body mass index (continuous variable).

^a^For analysis of the 1994–2001 period, 6,765 employees (378 employees who developed type 2 diabetes by 1993 and 661 employees who dropped out by 1993 were excluded).

^b^For analysis of the 2002–2009 period, 5,020 employees (799 employees who developed type 2 diabetes by 2001 and 1,985 employees who dropped out by 2001 were excluded).

**Figure 3.**
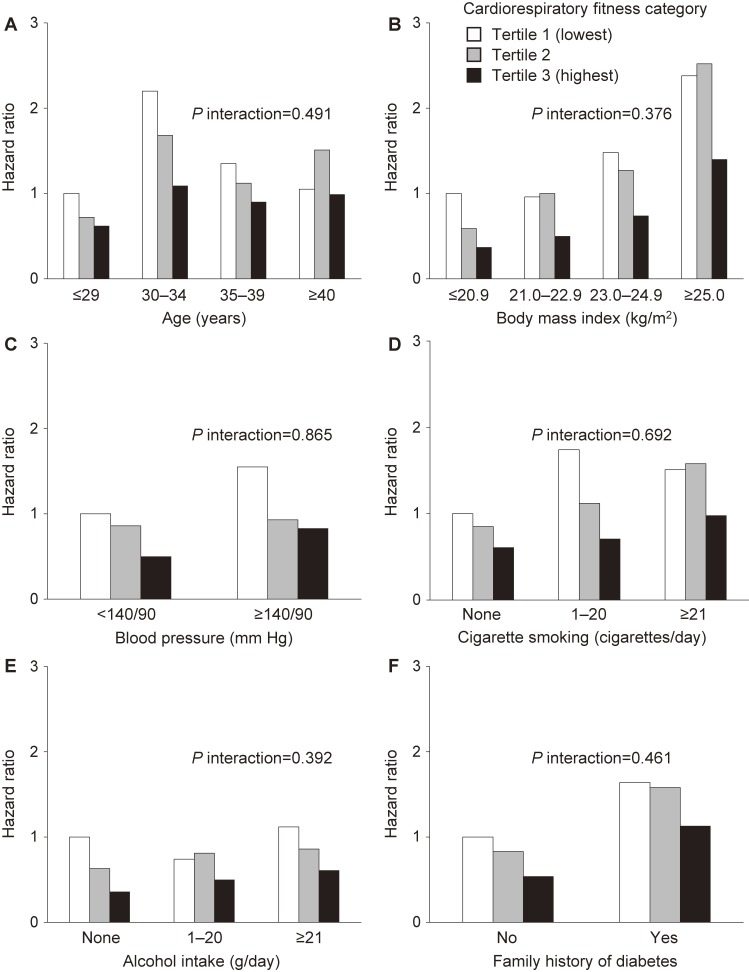
Multivariable-adjusted hazard ratios for incidence of type 2 diabetes according to cardiorespiratory fitness category in analysis stratified by potential risk factors (age (A), body mass index (B), blood pressure (C), cigarette smoking (D), alcohol intake (E), and family history of diabetes (F)), 2002–2009 (*n* = 5,020). Adjusted for all items in the figure.

To evaluate whether CRF and type 2 diabetes were related independently within other risk factor groups, even a long period of time after starting follow-up, we performed stratified analyses using potential risk factors for type 2 diabetes and calculated the hazard ratios for type 2 diabetes incidence for CRF tertiles (Figure 3). Even when employees aged ≥40 years were excluded, high CRF still exhibited a tendency to be associated with low incidence of type 2 diabetes regardless of the presence of potential risk factors (BMI, blood pressure, smoking habit, drinking habit, and family history of diabetes) and even when a long period of time had passed after starting the follow-up. There was no interaction between CRF (categorical variable) and each risk factor (categorical variable) on the incidence of type 2 diabetes.

## DISCUSSION

In the present prospective cohort study, the association between CRF and type 2 diabetes incidence was examined longitudinally by following the incidence of type 2 diabetes in Japanese men with an annual health checkup over a maximum of 23 years. This study showed that higher CRF was associated with lower risk for the development of type 2 diabetes in Japanese men. This inverse association was present over short and long follow-up periods, and magnitudes of association were similar.

Many previous prospective cohort studies indicated the association between CRF and type 2 diabetes incidence.^2^^–^^9^ The present study confirmed the association of higher CRF with a lower risk of type 2 diabetes incidence. There was no tendency of the association to weaken, even when the follow-up period was extended over a long time. Moreover, CRF was associated with type 2 diabetes incidence independent of various potential risk factors, even over an extended period.

CRF is known to decrease with age.^12^^,^^13^ On the other hand, CRF can be increased even in middle age via exercise training. A meta-analysis targeting randomized controlled trials indicated that the absolute and relative values of CRF increase with exercise training.^14^ Moreover, previous longitudinal studies examining the association of CRF due to changes in daily physical activities indicated that the degree of change in vigorous physical activity is directly related to CRF.^26^ Therefore, CRF can be considered to change in many ways, not only due to aging but also because of intentional or unintentional changes in the lifestyle and living environments, including physical activities. Previous studies examining the relationships between changes in CRF and the incidence of type 2 diabetes indicated an association between changes in CRF level and the incidence of type 2 diabetes. Previously, we reported that there is an association between the change in CRF over 7 years and subsequent incidence of type 2 diabetes in Japanese men.^16^ Moreover, according to the Coronary Artery Risk Development in Young Adults study, the rate of change in CRF for 7 years estimated using exercise duration with the modified Balke protocol was found to be associated with the incidence of type 2 diabetes for both men and women.^15^ The rate of decrease in CRF for persons with the incidence of type 2 diabetes compared to those without diabetes over two decades was reported to be higher. From the above findings, the influence of CRF level at baseline can be estimated to reduce the subsequent type 2 diabetes incidence due to the impact of aging and changes in lifestyle habits and living environments.

This is the first longitudinal study to include the follow-up period for CRF level at baseline and the subsequent period up to which CRF is associated with the incidence of type 2 diabetes. In the present study, the entire follow-up period was divided into three follow-up periods, ie, 7 years from baseline (1986–1993), 8 years between 7 and 15 years from baseline (1994–2001), 8 years between 15 and 23 years from baseline (2002–2009). The association between CRF at baseline and the incidence of type 2 diabetes was compared and examined for each of the three follow-up periods. The results showed that the risk of developing type 2 diabetes for the highest category of CRF compared with the lowest category of CRF at baseline for each of the follow-up periods was 0.69 times for the first 7 years from the baseline, 0.57 times in the next 8 years, and 0.47 times over the last 8 years. These observations indicated a clear inverse association between baseline CRF and the incidence of type 2 diabetes, regardless of the length of the follow-up period, and the association between CRF at baseline and incidence of type 2 diabetes did not weaken with a longer follow-up period. That is, these observations suggested that the CRF level may influence the subsequent incidence of type 2 diabetes over an extended period.

Major potential risk factors associated with the incidence of type 2 diabetes in addition to CRF include aging,^27^ obesity,^28^ high blood pressure,^29^ smoking^30^ and drinking habits,^31^ and family history of diabetes.^32^ In the present study, stratified analyses in 2002–2009 based on other risk factors were conducted. As a result, there was no interaction between CRF and each risk factor on the incidence of type 2 diabetes. The results suggested that CRF is still associated with type 2 diabetes, regardless of other risk factors, after a long period of time had passed from the start of the follow-up.

Several studies have examined the relationship between CRF and the incidence of type 2 diabetes for a long follow-up period. A significant association was not observed between CRF and the incidence of type 2 diabetes in the Kuopio Ischaemic Heart Disease study that conducted a follow-up with a median of 23 years for middle-aged men between 42 and 60 years old in Finland.^11^ However, the association of CRF at baseline with the incidence of type 2 diabetes for a subsequent extended period of over 2 decades or more has been reported by studies that followed 18-year-olds enlisted in the Swedish army for an average of 25.7 years follow-up (maximum 44 years follow-up),^9^ and Japanese male athlete university students with a median follow-up period of 26 years.^7^

The mechanism underlying the association by which CRF level at any given time influences incidence of type 2 diabetes over an extended period is not known, but epigenetic changes, called metabolic memory, may be involved. Some recent reports suggested that the exposure of metabolic functions to specific environmental factors in a given period may be stored in the body for an extended period and continue to affect subsequent metabolic regulation.^17^ Thus, it is possible that exposure of metabolic function in humans to a high CRF in a period may be retained within the cell as metabolic memory, and work prophylactically on the incidence of type 2 diabetes over an extended period. Another reason may be the involvement of genetic factors. The heritability of CRF was reported to be 25% to 65%,^33^ and persons with beneficial genetic background may maintain a relatively high CRF.

There were several limitations to the present study. First, the present study was conducted in Japanese males, and whether there are similar associations for women and other ethnicities is still not known. However, as many earlier studies in women and other ethnicities have confirmed the association between CRF and the incidence of type 2 diabetes, an association similar to that in the present study seems plausible. Second, changes in CRF during the follow-up period was not examined in the present study. Further studies regarding these changes in CRF are required. Finally, we controlled for the major potential risk factors for type 2 diabetes; however, some residual and unmeasured confounding may still remain in this observational study.

In conclusion, a high CRF is associated with a low incidence of type 2 diabetes over an extended period, regardless of other potential risk factors among Japanese men.
